# Prevailing Opinions on Connected Health in Austria: Results from an Online Survey

**DOI:** 10.3390/ijerph13080813

**Published:** 2016-08-11

**Authors:** Daniela Haluza, Marlene Naszay, Andreas Stockinger, David Jungwirth

**Affiliations:** Institute of Environmental Health, Center for Public Health, Medical University of Vienna, Kinderspitalgasse 15, Vienna A-1090, Austria; marlene.naszay@aon.at (M.N.); astockinger@me.com (A.S.); davidjungwirth@gmx.at (D.J.)

**Keywords:** Internet, health information, telemedicine, medical informatics, health education, eHealth, digital divide

## Abstract

New technological developments affect almost every sector of our daily lives, including the healthcare sector. We evaluated how connected health applications, subsumed as eHealth and telemedicine, are perceived in relation to socio-demographic characteristics. The current cross-sectional, online survey collected self-reported data from a non-probability convenience sample of 562 Austrian adults (58.9% females). The concept of eHealth and telemedicine was poorly established among the study population. While most participants already used mobile devices, they expressed a quite low desirability of using various telemedicine applications in the future. Study participants perceived that the most important overall benefits for implementing connected health technology were better quality of healthcare, location-independent access to healthcare services, and better quality of life. The respective three top-ranked overall barriers were data security, lack of acceptance by doctors, and lack of technical prerequisites. With regard to aging societies, healthcare providers, and users alike could take advantage of inexpensive, consumer-oriented connected health solutions that address individual needs of specific target groups. The present survey identified issues relevant for successful implementation of ICT-based healthcare solutions, providing a compilation of several areas requiring further in-depth research.

## 1. Introduction

In the current digital information era, high penetration of Internet and smartphone use enables technology-based health communication activities and public health interventions [1]. As for terminology, the concepts of eHealth and telemedicine are used rather inconsistently for denoting the use of information and communication technologies (ICT) to support healthcare delivery [2,3]. In a broader sense, eHealth refers to Internet-enabled health services and information delivery, whereas telemedicine characterizes “tele”-labeled healthcare services, such as telemonitoring and telecare, using ICT for exchanging medical information [2,4,5,6]. Further, the notion connected health combines both concepts of eHealth and telemedicine and is used as the umbrella term for the according sociotechnical perspective linking people, processes, and technology in healthcare in an increasingly interconnected world [7,8].

Applying remote medical diagnosis and monitoring based on mobile health systems can significantly reduce healthcare costs and increase the quality of healthcare delivery by facilitating disease management. Notwithstanding the apparent promise of connected health applications to reduce healthcare inequalities, health information technologies have proved difficult to implement [9]. Designated implementation barriers include potential threats to confidentiality and privacy, lack of system interoperability with electronic health records and other IT tools, decrease in face to face communication between doctor and patient, and device and sensor type-specific difficulties [10,11].

Diffusion of eHealth and telemedicine ultimately depends on its acceptance among health professionals and the general population alike [12]. Current findings gathered in an Austrian Delphi survey indicated divergent perceptions and expectations regarding connected health services among the healthcare stakeholder groups health professionals, patient advocates, and administrative personnel [13,14]. Despite nearly ubiquitous Internet access, digital divide factors, such as age, gender, and education, predict adoption of health information technologies. Thus, Prestin et al. suggest documenting socio-economic factors influencing national prevalence, trends, and user profiles of online health activities [15]. To address the dynamics of recent technological developments in healthcare delivery, knowledge on up-to-date public perceptions to understand interactions between involved social, technical, and human factors is essential. Thus, the current study investigated prevailing views on eHealth and telemedicine and respective effects of socio-demographic characteristics [16]. The main objective of this study was to provide the hitherto lacking baseline evidence to further identify trends and new developments in national technology-assisted healthcare in a subsequent larger-scale study among the general Austrian population. Noted health outcome disparities across social groups argue for examining socio-demographic differences in implementation, uptake, and impact of connected health strategies [17]. Thus, identifying the extent of differences in connected health adoption based on socio-demographic characteristics offers important implications for implementation research and practice.

## 2. Materials and Methods

### 2.1. Study Design

This cross-sectional study assessed knowledge, awareness, and perceptions regarding eHealth and telemedicine among a non-probability convenience sample of Austrian adults. The study was approved by the ethics committee of the Medical University of Vienna (No. 1303/2015) and performed in accordance with the principles of the Declaration of Helsinki. We created an online survey with SoSci Survey Version 2.5.00-i (SoSci Survey GmbH, Munich, Germany) [18,19]. The survey was open and accessible to respondents on www.soscisurvey.com from August to October 2015. As the survey software prevented item non-response, we collected fully completed questionnaires without missing values. The cover page of the questionnaire specified the research background and objectives of the study, as well as the academic and anonymous nature of the survey. Only persons from within the research group were authorized to access collected data.

### 2.2. Participants’ Recruitment

We followed a self-selection snowball recruiting strategy in four phases to enroll a minimum sample size of 500 participants, achieving a research power of at least 80% (alpha = 0.05). This snowball sampling approach through referral by friends as well as newsgroups and Internet forum websites has proven effective for recruiting survey participants [20]. Additionally, the virtual response rate via social media excels traditional survey techniques [21]. Thus, we e-mailed invitation letters asking respondents to complete the survey via a hyperlink. For that, we used mailing lists of health-related professional associations in phase 1, followed by distribution of the link via social media (i.e., facebook) in phase 2. In phase 3, we invited participants via online health forum homepages in German. In phase 4, we sent reminder e-mails every two weeks after the initial invitation e-mail to prompt further completions. To accomplish the snowball sampling effect in all phases, we asked study subjects to also distribute this invitation e-mail among their personal contacts. The survey was closed another two weeks after sending out the final reminder e-mails.

### 2.3. Study Questionnaire

A structured questionnaire in German was developed based on previous research work [13,14]. We carried out a paper and pencil-based pilot survey among 20 employees and medical students of the Medical University of Vienna to validate the questionnaire and assess its general comprehensibility. We collected self-reported information on age, gender, place of living by Austria´s geographic regions (capital Vienna, East, and West), education (primary, secondary, and tertiary), and health profession, as well as using mobile devices and occurrence of chronic disease (all: yes/no). We dichotomized education level (no university, i.e., primary and secondary education/university, i.e., tertiary education) to build the variable education.

Previously, in 2001, Prensky described differences between individuals who grew up in the digital world (digital natives) and those who did not, but have adopted the emerging postmillennial digital culture (digital immigrants). This terminology is widely applied in research aiming at understanding differences between digital generations [22,23]. Prensky further characterized the typical birthdate for digital native status as 1980 and after [16]. Thus, in our study, participants aged 35 years and younger formed the group of digital natives and those aged 35+ the group of digital immigrants.

In order to align the individual level of knowledge among participants, we stated the according definitions of eHealth and telemedicine at the beginning of the respective questionnaire sections [2,4,5]. We used the nomenclature most commonly used in Austria, and also approved in the aforementioned pilot survey. We defined eHealth as health services and information delivered or enhanced through the Internet and related technologies in the interface of medical informatics, public health, and business [6]. Further, we referred to telemedicine for healthcare services such as telemonitoring and telecare using ICT for exchanging medical information [2,4,5].

Rank order questions allowed study subjects to specify how items or concepts stacked up against each other. As for, participants used a simple drag and drop feature to rank potential benefits and barriers for eHealth as well as telemedicine implementation in two different items. For analytical purposes, we consolidated these items to build the general construct of connected health. The selectable choices were borrowed from the literature. To assess importance of specific benefits, we used the wording “The application of eHealth/telemedicine may implement certain benefits. Please rank the following benefits according to their importance.” The seven choices offered were better quality of life, better quality of healthcare, better financing of healthcare, avoidance of multiple diagnostic tests, better relationship between doctors and patients, increasing knowledge of patients, and location-independent access to healthcare services [8,10,12,13,24,25]. As for barriers, we used the wording “The application of eHealth/telemedicine can be hampered by certain factors. Please rank the following hampering factors according to their importance.” The six choices offered were costs/financing, data security, lack of acceptance by doctors, lack of acceptance by patients, increase of administrative burden, and lack of technical prerequisites [4,8,10,13,14,26,27,28,29].

We assessed self-reported perceived levels of eHealth and telemedicine knowledge by asking “How well informed do you feel about eHealth?” and “How well informed do you feel about telemedicine?” The according variables were eHealth knowledge and telemedicine knowledge, respectively [4]. We measured perceived reliability of online health information by asking “In general, how would you assess the reliability of health information from the Internet?” building the variable reliability health information. Furthermore, we evaluated perceived reasonability of data exchange between doctors and patients by asking “How would you rate the reasonability of electronic health information exchange between healthcare professionals and patients?” The according variable was reasonability data exchange [24]. We asked participants to indicate their agreement with the two statements “Permanently collecting health data or behavior via mobile devices for monitoring a chronic disease is useful” and “Permanently collecting health data or behavior via mobile devices to receive personalized lifestyle recommendations is useful.” The according variables were desirability monitoring and desirability lifestyle, respectively.

### 2.4. Data Analysis

Median splitting dichotomized the five-point Likert scales ranging from very poor (=1) to very good (=5) for the variables eHealth and telemedicine knowledge, reliability health information, reasonability data exchange, desirability monitoring and lifestyle (all: low/high scores). Using descriptive statistics, we reported qualitative values in percentages, means, and standard deviations (SD). Regarding the ranking questions, the average ranking of each item indicated which aspect was most important to participants. We were especially interested in the three top-ranked items of all categories. With “1” indicating the top rank, the item with the lowest mean was ranked highest. Mann-Whitney U tests compared choices of benefits and barriers for connected health, eHealth, and telemedicine across digital age group, gender, health profession, and education. Chi^2^ tests analyzed respective group-specific differences on perceived eHealth and telemedicine knowledge, reliability health information, reasonability data exchange, as well as desirability monitoring and lifestyle.

Binary logistic regression models measured the relationship between the categorical dependent and independent variables by estimating probabilities. The dichotomized dependent variables were eHealth and telemedicine knowledge, reliability of health information, reasonability of data exchange, desirability monitoring, and desirability lifestyle (all: low vs. high). The dichotomized independent variables were digital age group (digital natives vs. digital immigrants), gender (females vs. males), education (non-university vs. university), and health profession (yes vs. no). We selected the models best fitting to the data as indicated by Nagelkerke’s R^2^ and log likelihood tests. We calculated crude and adjusted odds ratios (OR) and 95% CI and reported results of the adjusted regression models controlled for the variables using mobile devices, chronic disease, and place of living (all: yes vs. no). All statistical analyses were conducted in IBM SPSS Statistics Version 22 (IBM Co., Chicago, IL, USA). Two-sided level of significance was set to *p* < 0.05.

## 3. Results

### 3.1. Socio-Demographic Characteristics

Table 1 summarizes the socio-demographic characteristics of the study population and the according Austrian census data from 2011 [30]. Of all unique visitors of the online questionnaire (*n* = 1201), 77% of respondents fully completed the questionnaire (*n* = 562, 58.9% females, mean age 36.9 ± 15.2). We did not find statistically significant differences between the total sample and participants with partially completed questionnaires, but full information on socio-demographic data regarding age, gender, and health profession. Of all study subjects, 54.3% represented the group of digital natives and 43.2% the health profession group. Exactly half of the respondents reported primary and secondary education combined, whereas the other half already finished university (tertiary). Further, 56.9% of study subjects suffered from at least one chronic condition, 94.7% used mobile devices, and 63.5% lived in Vienna, the capital of Austria. In comparison to the general Austrian population, study subjects were more likely to live in the capital Vienna, to be female, to be better educated, and to use mobile devices.

### 3.2. Perceived Benefits and Barriers

Table 2 shows the distribution of rankings for connected health-related benefits and barriers. As for benefits, location-independent access to healthcare services achieved the highest proportion for rank 1 (26.6%), and better financing of healthcare (3.2%) the lowest. Data security achieved the highest proportion for rank 1 (54.1%); increase of administrative burden (3.7%) the lowest in the category barriers. Further, based on the average ranking of each item indicated by the mean, we determined the overall item rank (Table 3). The three top-ranked respective benefits were better quality of healthcare, location-independent access to healthcare services (with statistically significant results for gender and education), and better quality of life (with statistically significant results for all groups except health professional). The three top-ranked barriers were data security (with statistically significant results for digital age group), lack of acceptance by doctors, and lack of technical prerequisites (both statistically significant results for gender).

We further collected opinions on benefits and barriers for eHealth as well as telemedicine implementation separately and assessed differences between socio-demographic groups (Table 4). The three top-ranked benefits regarding eHealth were better quality of healthcare, increasing knowledge of patients, and avoidance of multiple diagnostic tests (with statistically significant opinions only for the latter within digital age group and health profession). Data security, lack of acceptance by doctors, and lack of acceptance by patients were the top-ranked respective barriers (with statistically significant divergent opinions only for acceptance by doctors within digital age group and gender). The top-ranked benefits regarding telemedicine were location-independent access to healthcare services, better quality of healthcare, and better quality of life (with statistically significant divergent group opinions for all groups except health profession regarding the first and third factor). Respective top-ranked barrier were data security (with statistically significant differences for digital age group), costs/financing (without any intergroup differences), and lack of acceptance by doctors (with statistically significant gender differences).

We analyzed differences across digital age group, gender, health profession, and education in six domains relevant to health technology adoption, i.e., eHealth and telemedicine knowledge, reliability health information, reasonability data exchange, as well as desirability monitoring and lifestyle scores (Table 5). Participants mostly perceived their eHealth and telemedicine knowledge, as well as reliability of online health information, as moderate to poor (35.1% and 35.8%, 27.0% and 37.5%, 23.3%, and 47.9%, respectively). Reasonability of data exchange was mostly rated as good (41.5%) and moderate (44.7%). We found dissenting views on desirability monitoring and lifestyle with ratings ranging from good to very poor, hence with a trend towards more favorable attitudes regarding desirability monitoring. We revealed statistically significant differences in group-specific views: gender and health profession on perceived eHealth knowledge; digital age group, gender, and health profession on telemedicine knowledge; digital age group on reliability; gender on reasonability data exchange; digital age group and education on desirability monitoring; and digital age group on desirability lifestyle.

Using six separate binary logistic regression analyses, we scrutinized the reported socio-demographic differences regarding eHealth and telemedicine knowledge, reliability health information, reasonability data exchange, desirability monitoring and lifestyle (dependent variables). All models showed a good fit to the data according to goodness of fit measures. Only results of the adjusted models are presented in Table 6. eHealth knowledge was predicted by health profession with non-health professionals being 40% less likely to report high respective knowledge (OR = 0.60, 95% CI 0.39–0.91), education with having an university degree accounted for 64% higher odds (OR = 1.64, 95% CI 1.07–2.51), and telemedicine knowledge (OR = 12.60, 95% CI 8.01–19.83). Telemedicine knowledge was predicted by female gender (OR = 1.92, 95% CI 1.25–2.96), health profession (OR = 0.56, 95% CI 0.37–0.87), and eHealth knowledge (OR = 12.61, 95% CI 8.01–19.85). Further, predictors for reliability health information included digital age group with digital immigrants being 44% more likely to perceive online information as reliable (OR = 0.56, 95% CI 0.38–0.82) and reasonability data exchange (OR = 0.60, 95% CI 0.41–0.89). The latter factor was predicted by reliability health information, desirability monitoring, and desirability lifestyle. Desirability monitoring was predicted by digital age group with digital immigrants being 51% more likely to find online information reliable (OR = 0.56, 95% CI 0.31–0.75) as well as reasonability data exchange and desirability lifestyle (both: *p* < 0.001). Finally, desirability lifestyle was predicted by digital age group, with digital immigrants being 40% more likely to find lifestyle monitoring desirable (OR = 0.60, 95% CI 40–0.90), and by gender, with females being less likely to do so (OR = 0.57, 95% CI 0.39–0.84), and desirability monitoring (*p* < 0.001).

## 4. Discussion

In light of aging societies and ICT-driven paradigm shifts in healthcare, evidence on inequalities in access to and usage of constantly evolving technologies is relevant for researchers, decision-makers, and healthcare providers alike [31]. Whereas these aspects of health services research are already studied in other countries, little knowledge is available for the general Austrian population so far [4,11,12,20,26,27,28,29]. To examine prevailing perceptions and expectations regarding health technology use, we investigated views on connected health and according socio-demographic differences within the context of Austria. Moreover, this hypothesis-generating, cross-sectional study served as a feasibility study providing the methodological basis to conduct a larger-scale survey among a nationally representative study sample.

In the current study, participants perceived that the three top-ranked benefits of connected health were better quality of healthcare, location-independent access to healthcare services, and better quality of life. The three respective top-ranked barriers were data security, lack of acceptance by doctors, and lack of technical prerequisites. The option of better quality of healthcare, which ranked at the top in our study, was also ranked among the top three advantages of future ICT solutions by Austrian healthcare experts [14]. The power of connected health applications to enhance the quality of healthcare, in general, might be assigned to the combined effects of increasing efficiency of healthcare delivery and doctor-patient communication and thus, reducing adverse patient outcomes [28,29,32].

We ascertained that participants shared the same knowledge by providing a commonly accepted definition of the terms eHealth and telemedicine in the study questionnaire [2,4,5,6]. The most important benefits were better quality of healthcare for eHealth implementation, and location-independent access to healthcare services for telemedicine implementation. Thus, the analysis of benefits and barriers related to eHealth and telemedicine implementation separately clearly reflected the different inherent meaning of these terms. Notwithstanding that these two concepts are highly interrelated and potentially interchangeable for laypersons, study subjects differentiated between the underlying concepts and the potential impact on themselves. We further revealed several statistically significant differences in socio-demographic attributes, substantiating the divergent perceptions of associated benefits and barriers. Given the challenges of defining these constantly evolving terms, we suggest adequate consumer-centric public information and education for all strata of end user on a regular basis [2].

The result on data security being perceived as most important barrier suggests objections regarding data security and privacy, and is in line with several studies [12,13,14,29,33]. Computerized health data that could be potentially accessed by hackers, as well as insurance companies, have elevated public concerns about potential privacy and security violations, introducing the challenge of addressing ethical and legal barriers towards connected health [24,26,27]. Thus, addressing aspects of privacy, confidentiality, and data security is vital for successful eHealth and telemedicine implementation [34]. Noteworthy, privacy and confidentiality of information can be seen as both facilitators and barriers to connected health adoption [35]. Remote patient monitoring could protect privacy by allowing people to be monitored from home, thus offering more independence, while providing personal health information over the Internet could likewise intrude upon individual privacy. In contrast, Barr et al. found limited public concerns regarding potential privacy threats of personal information storage and monitoring device use [8]. In our survey, the majority of participants regularly used mobile devices such as smartphones. Additionally, literature suggests that patients are willing to renounce on some aspects of their privacy if connected health services provide other benefits, e.g., increased independence for the elderly [34,36].

Study participants perceived the lack of technical prerequisites as one of the top-ranked barriers for implementing connected health services. This finding is in line with other authors reporting that lack of competence and equipment hampers connected health implementation [28,29]. Additionally, usability deficiencies such as system failures and lacking integration of these systems have been shown to reduce efficiency of clinical ICT use and hampered physician's routine work [37]. In this vein, lack of acceptance by doctors was ranked among the three most important hampering factors, supporting findings of other authors [14,29]. In general, interest in using eHealth and telemedicine services is most strongly associated with technology confidence and perceived advantages and disadvantages among physicians [38]. Furthermore, doctors have been shown to be rather skeptical regarding innovative technologies compared to other health-related professionals [13,14]. These encounters are of relevance as physicians could act as hub by assisting patients in gaining confidence using technologies through both highlighting benefits and addressing their concerns [39,40]. However, in the current study, being a health professional did not account for a stronger refusal compared to other socio-demographic variables. Further research could objectively study the impact of successful connected health-related implementations on patient, provider, and economic outcomes [13,14,25]. Hospital training programs for all doctors, as well as awareness programs for patients, are required to stimulate future eHealth and telemedicine utilization [41].

Research supports the theory of a divide between different generations regarding their approach towards using computer-based new media [42,43]. Some of the associated challenges include that a new generation of digital natives are currently in their pursuit of a medical education, requiring faculty members to become digital immigrants [44]. Similar aspects of generational digital divide apply to the doctor-patient relationship in everyday counseling. In our survey, digital age influenced telemedicine knowledge, reliability health information, as well as desirability monitoring and lifestyle. Gender aspects were relevant for eHealth and telemedicine knowledge, and reasonability data exchange. Health profession influenced eHealth and telemedicine knowledge, and education desirability monitoring. Regression analyses scrutinized these group-specific effects. In regard to digital age groups, digital immigrants were more likely to find online health information as reliable, as well as disease monitoring and lifestyle monitoring as desirable.

As for gender differences, female gender predicted the variable telemedicine knowledge and male gender predicted desirability lifestyle. eHealth and telemedicine knowledge were predicted by health profession, with a higher education level additionally predicting eHealth knowledge. These results are in agreement with other studies reporting significant differences in eHealth and telemedicine use related to socio-economic status, age, and gender [8,17]. In a Canadian study, a lack of knowledge about telemedicine, time constraints, and funding were the three most important factors hampering connected health implementation in clinical settings [4]. In comparison to younger age groups, those aged over 60 years were shown to be less willing to use connected health [8]. Kontos et al. found that elderly, male US adults with lower socio-economic status were less likely to engage in eHealth activities [8,17]. Patients with lower levels of education had significantly lower odds of using the Internet to communicate with a doctor, track their personal health information, diet, weight, and physical activity online, or download health information to a mobile device. Additionally, females were more likely to use eHealth across healthcare and user-generated content and sharing domains, whereas age was primarily influential for health information-seeking.

Overall, study participants expressed a by trend low desirability of using various connected health applications in the future. Participants mostly perceived their eHealth and telemedicine knowledge, as well as reliability of online health information as moderate to poor, and reasonability of data exchange was rated as good to moderate. We found dissenting views on desirability monitoring and lifestyle with ratings ranging from good to very poor. Whereas study participants expressed cautious respective views, Simon et al. reported that patients were enthusiastic about electronic health information exchange, recognizing its capacity to improve the quality and safety of healthcare [24]. However, electronic health information exchange could open the door for breached privacy and health data misuse [24]. Whiddett et al. found that patients in New Zealand are skeptical regarding sharing personal medical information and, moreover, would like to be informed about current information-sharing practices [45]. As the exchange of electronic health information becomes more widespread, users should have access to concise educational materials and opportunities to engage in conversations about associated benefits and risks. Future systems should also incorporate sophisticated and flexible access control policies that can be adapted to individual user preferences [45]. These considerations argue for intensified pre- and post-implementation research on health technology approval in the context of existing models of technology acceptance [43].

eHealth and telemedicine interest is most strongly associated with technology confidence and perceived benefits and barriers [39]. In line with Edwards et al., our data suggest that helping patients gain confidence in using health technologies, highlighting benefits, and addressing concerns could decrease the skeptic attitude towards connected health among the general population [39]. Respective recommendations for policy-makers, developers, and health professionals include tailoring services to meet the needs of a broad range of users, increase their personal responsibility, and underpin the role of health professionals for promoting and facilitating the adoption of eHealth services [46]. Further research endeavors should investigate aspects relevant for realizing the benefits of connected health such as evidence of cost-effectiveness, development of user profile-specific applications, incentives for health professionals, liability issues, and end user IT skills [9].

### Study Limitations

The results of this study should be considered within the context of its design and the associated limitations. The scope of this feasibility study was restricted to a small sample of Austrian citizens [47]. We, therefore, did not claim that the results were generalizable to the general Austrian population, which is also obvious when comparing the respective socio-demographic characteristics depicted in Table 1. Especially, our study sample was more likely to live in the capital Vienna, to be female, and be better educated. However, the primary purpose of this hypothesis-generating, cross-sectional study was to determine whether the study questionnaire was sufficiently accurate to warrant an expanded study, and to test whether the snow-ball sampling design of an online survey was accepted by participants.

Web-based surveys are one of the most widely utilized survey methods that facilitate low-cost, fast data collection from the target population [48]. Advantages of online surveys also include increased response rates due to convenient, place- and time-independence and low-threshold participation for respondents, automatized data input and handling with a smaller possibility of data errors. However, Internet surveys lead to so-called non-probabilistic samples due to the inherent anonymity and selection processes of the snowball sampling. As we considered the benefits of the online survey to outweigh the potential weaknesses, we suggest that this methodological approach was adequate for conducting a feasibility survey among a sub-sample of the general population.

Given the lack of administrative data, we collected self-reported data introducing survey response bias. Additionally, Internet users might not fully represent the general population, reducing generalizability of the study results. Using a survey questionnaire in German might have introduced a language bias. Nevertheless, the purpose of this study was to assess a snapshot of prevailing views on eHealth and telemedicine in the context of Austria’s well-funded healthcare system [49]. As such, it is likely to have identified some relevant topics of general interest for healthcare decision-makers and the interested research community, thus initiating dialogue and debate in this steadily evolving field.

Our findings on different views on connected health across social groups warrant further in-depth research in this area including exploring predictors of health consumers' technology adoption such as health literacy and Internet access [1]. Due to the lack of pre-existing reference data, it is not clear whether these results indicated a narrowing or widening divide in views on connected health across population groups. In particular, no projection can be made in regard to the respective situation of medically underserved and disadvantaged individuals. In the future, clinical care and public health communication efforts should acknowledge different eHealth and telemedicine usage to better address communication inequalities and persistent health disparities whenever possible [17].

## 5. Conclusions

The current study provides so far lacking knowledge on views on connected health potentially relevant for identifying aspects that should receive attention when implementing according national, target group-tailored healthcare and public health promotion strategies. Overall, study participants were cautious regarding reliability of online health information, reasonability of data exchange, and desirability of using connected health applications in the future. Additionally, participants mostly perceived their eHealth and telemedicine knowledge as improvable. Whether the reported findings on different perceptions among socio-demographic groups indicated early evidence of a narrowing divide in eHealth and telemedicine use across population groups as a result of the ubiquitous Internet access and computer ownership warrants further exploration. From a health services research perspective, these results emphasize the need to investigate prevailing use and expectations regarding eHealth and telemedicine tools, in particular among different strata of the population, such as medically underserved and disadvantaged groups.

## Figures and Tables

**Table 1:** Socio-demographic characteristics of the study population and the Austrian census data from 2011.

	Total		Austrian Census Data
	N	%	%
**Total**	562	100	100
**Gender**			
Female	331	58.9	51.3
Male	231	41.1	48.7
**Place of living**			
Capital Vienna	357	63.5	20.4
East	133	23.7	43.6
West	72	12.8	36.0
**Chronic disease**			
Yes	320	56.9	62.3
No	242	43.1	37.7
**Using mobile devices**			
Yes	532	94.7	84.0
No	30	5.3	16.0
**Age groups (years)**			
<29	261	46.4	41.4
30–39	82	14.6	13.1
40–49	70	12.5	16.5
50–59	99	17.6	13.6
>60	50	8.9	15.4
**Digital age group**			
Digital natives (<35 years)	305	54.3	54.5 ^§^
Digital immigrants (>35 years)	257	45.7	45.5
**Education level**			
Primary	90	16.0	19.2
Secondary	191	34.0	65.1
Tertiary	281	50.0	15.7
**Education**			
No university (primary and secondary)	281	50.0	84.3
University (tertiary)	281	50.0	15.7
**Health profession**			
Yes	243	43.2	-
No	319	56.8	-

Note: ^§^ Age group < 40 years.

**Table 2:** Overall distributions of ranking regarding connected health-related benefits and barriers.

	Overall Rank (%)							
	1	2	3	4	5	6	7	Total
**CONNECTED HEALTH**								
**Benefits**								
Better quality of life	21.4	13.4	11.7	14.9	14.3	11.8	12.7	100
Better quality of healthcare	17.6	21.6	15.1	16.4	11.3	12.5	5.6	100
Better financing of healthcare	3.2	10.6	15.2	13.5	14.0	19.2	24.5	100
Avoidance of multiple diagnostic tests	12.8	16.9	14.9	12.9	14.2	14.3	14.2	100
Better relationship between doctors and patients	3.3	8.1	13.8	14.5	19.8	19.1	21.5	100
Increasing knowledge of patients	15.2	13.7	17.3	17.5	15.8	12.5	8.3	100
Location-independent access to healthcare services	26.6	16.0	12.1	10.4	10.9	10.8	13.5	100
**Barriers**								
Costs, financing	12.9	15.3	16.8	17.4	19.2	18.6	-	100
Data security	54.1	17.0	9.8	7.2	6.0	6.0	-	100
Lack of acceptance by doctors	10.1	18.1	23.7	19.0	17.9	11.4	-	100
Lack of acceptance by patients	7.5	20.3	19.6	18.3	17.3	17.2	-	100
Increase of administrative burden	3.7	10.3	13.5	22.3	25.4	24.9	-	100
Lack of technical prerequisites	11.9	19.1	16.8	16.0	14.4	22.1	-	100

**Table 3:** Ranking of connected health-related benefits and barriers, ordered by total rank.

	Total			Digital Age Group	Gender	Health Profession	Education
	Mean	SD	Rank			*p* Values	
**CONNECTED HEALTH**							
**Benefits**							
Better quality of healthcare	3.4	1.6	1	0.537	0.139	0.744	0.715
Location-independent access to healthcare services	3.5	1.9	2	0.064	0.002 *	0.629	0.011 *
Better quality of life	3.7	1.9	3	0.001 **	0.013 *	0.405	0.023 *
Increasing knowledge of patients	3.8	1.5	4	0.086	0.120	0.017 *	0.456
Avoidance of multiple diagnostic tests	4.0	1.7	5	0.001 **	0.462	0.032 *	0.390
Better financing of healthcare	4.8	1.6	6	0.203	0.019 *	0.250	0.843
Better relationship between doctors and patients	4.8	1.5	7	0.478	0.406	0.847	0.019 *
**Barriers**							
Data security	2.1	1.4	1	0.038 *	0.068	0.680	0.178
Lack of acceptance by doctors	3.5	1.4	2	0.127	0.003 *	0.111	0.645
Lack of technical prerequisites	3.7	1.5	3	0.092	0.001 **	0.306	0.757
Lack of acceptance by patients	3.7	1.4	4	0.115	0.623	0.114	0.947
Costs, financing	3.7	1.5	5	0.254	0.053	0.614	0.093
Increase of administrative burden	4.3	1.3	6	0.204	0.769	0.136	0.917

Note: Mann-Whitney U tests * *p* < 0.05; ** *p* < 0.001.

**Table 4:** Ranking of benefits and barriers of eHealth and telemedicine, ordered by total rank.

	Total			Digital Age Group	Gender	Health Profession	Education
	Mean	SD	Rank			*p* Values	
**EHEALTH**							
**Benefits**							
Better quality of healthcare	3.4	1.8	1	0.056	0.673	0.961	0.813
Increasing knowledge of patients	3.5	1.9	2	0.900	0.502	0.065	0.295
Avoidance of multiple diagnostic tests	3.7	2.0	3	0.001 **	0.958	0.008 **	0.109
Location-independent access to healthcare services	3.8	2.1	4	0.344	0.050 *	0.280	0.049 *
Better quality of life	3.9	2.0	5	0.001 *	0.075	0.471	0.041 *
Better relationship between doctors and patients	4.8	1.7	6	0.207	0.155	0.586	0.004 *
Better financing of healthcare	4.9	1.8	7	0.830	0.101	0.504	0.568
**Barriers**							
Data security	1.9	1.4	1	0.913	0.332	0.862	0.232
Lack of acceptance by doctors	3.5	1.5	2	0.039 *	0.003 *	0.202	0.631
Lack of acceptance by patients	3.7	1.6	3	0.252	0.289	0.124	0.628
Lack of technical prerequisites	3.7	1.7	4	0.040 *	0.001 **	0.397	0.936
Costs, financing	3.9	1.6	5	0.754	0.057	0.894	0.201
Increase of administrative burden	4.2	1.5	6	0.111	0.174	0.121	0.523
**TELEMEDICINE**							
**Benefits**							
Location-independent access to healthcare services	3.2	2.1	1	0.017 *	0.001 **	0.866	0.015 *
Better quality of healthcare	3.4	1.8	2	0.463	0.037	0.565	0.642
Better quality of life	3.6	2.1	3	0.001 **	0.006 *	0.643	0.037 *
Increasing knowledge of patients	4.0	1.8	4	0.002 *	0.095	0.036 *	0.818
Avoidance of multiple diagnostic tests	4.2	1.9	5	0.001 **	0.192	0.227	0.741
Better financing of healthcare	4.7	1.8	6	0.063	0.019 *	0.199	0.995
Better relationship between doctors and patients	4.9	1.7	7	0.895	0.800	0.852	0.202
**Barriers**							
Data security	2.3	1.6	1	0.004 *	0.052	0.550	0.280
Costs, financing	3.5	1.7	2	0.090	0.105	0.459	0.076
Lack of acceptance by doctors	3.5	1.5	3	0.429	0.012 *	0.135	0.872
Lack of technical prerequisites	3.6	1.7	4	0.391	0.001 **	0.368	0.651
Lack of acceptance by patients	3.6	1.6	5	0.123	0.892	0.213	0.830
Increase of administrative burden	4.4	1.4	6	0.353	0.047 *	0.283	0.658

Note: Mann-Whitney U tests * *p* < 0.05; ** *p* < 0.001.

**Table 5:** Respondents’ views in relation to eHealth and telemedicine knowledge, reliability, and reasonability, as well as desirability monitoring and lifestyle.

	eHealth Knowledge	Telemedicine Knowledge	Reliability Health Information	Reasonability Data Exchange	Desirability Monitoring	Desirability Lifestyle
**Choices of answer; N (%)**						
Very good	11 (2.0)	12 (2.1)	20 (3.6)	13 (2.3)	49 (8.7)	30 (5.3)
Good	70 (12.5)	47 (8.4)	233 (41.5)	50 (8.9)	172 (30.6)	122 (21.7)
Moderate	197 (35.1)	152 (27.0)	251 (44.7)	131 (23.3)	107 (19.0)	115 (20.5)
Poor	201 (35.8)	211 (37.5)	54 (9.6)	269 (47.9)	124 (22.1)	149 (26.5)
Very poor	83 (14.8)	140 (24.9)	4 (0.7)	99 (17.6)	110 (19.6)	146 (26.0)
**Socio-demographic characteristics; *p* values**						
Digital age group	0.213	0.004 *	0.008 *	0.142	0.001 **	0.001 **
Gender	0.042 *	0.001 **	0.441	0.001 **	0.253	0.162
Health profession	0.001 **	0.001 **	0.699	0.395	0.318	0.125
Education	0.138	0.509	0.061	0.802	0.031 *	0.101

Note: Chi^2^ tests * *p* < 0.05; ** *p* < 0.001.

**Table 6:** Binary logistic regression analysis for variables predicting connected health-related dichotomized scores.

	eHealth Knowledge	Telemedicine Knowledge	Reliability Health Information	Reasonability Data Exchange	Desirability Monitoring	Desirability Lifestyle
	OR (95% CI)					
**Socio-demographic characteristics**						
Digital age group	0.96 (0.61; 1.52)	1.34 (0.84; 2.14)	0.56 (0.38; 0.82) *	1.04 (0.67; 1.60)	0.49 (0.31; 0.75) **	0.60 (0.40; 0.90) *
Gender	1.14 (0.75; 1.74)	1.92 (1.25; 2.96) *	1.02 (0.72; 1.46)	1.14 (0.76; 1.70)	1.22 (0.81; 1.83)	0.57 (0.39; 0.84) *
Health profession	0.60 (0.39; 0.91) *	0.56 (0.37; 0.87) *	1.25 (0.87; 1.79)	1.00 (0.67; 1.50)	1.37 (0.91; 2.07)	1.33 (0.90; 1.95)
Education	1.64 (1.07; 2.51) *	1.03 (0.66; 1.61)	1.08 (0.75; 1.55)	1.15 (0.77; 1.73)	0.71 (0.47; 1.07)	1.05 (0.72; 1.55)
**Scores ^&^**						
eHealth knowledge	Dependent variable	12.61 (8.01; 19.85) **	0.70 (0.46; 1.05)	1.33 (0.84; 2.09)	0.97 (0.61; 1.55)	1.24 (0.80; 1.92)
Telemedicine knowledge	12.60 (8.01; 19.83) **	Dependent variable	1.17 (0.77; 1.79)	1.20 (0.74; 1.94)	1.42 (0.88; 2.29)	1.05 (0.67; 1.65)
Reliability health information	0.70 (0.46; 1.05)	1.16 (0.76; 1.78)	Dependent variable	0.60 (0.40; 0.88) *	0.69 (0.46; 1.03)	0.99 (0.68; 1.44)
Reasonability data exchange	1.35 (0.86; 2.13)	1.17 (0.73; 1.88)	0.60 (0.41; 0.89) *	Dependent variable	3.46 (2.19; 5.45) **	1.54 (1.02; 2.32)
Desirability monitoring	0.98 (0.61; 1.56)	1.39 (0.86; 2.24)	0.71 (0.48; 1.05)	3.39 (2.16; 5.33) **	Dependent variable	4.27 (2.88; 6.35) **
Desirability lifestyle	1.26 (0.81; 1.95)	1.07 (0.68; 1.68)	0.99 (0.68; 1.44)	1.57 (1.04; 2.36) *	4.31 (2.90; 6.41) **	Dependent variable

Note: All models control for using mobile devices, chronic disease, and place of living. * *p* < 0.05; ** *p* < 0.001; ^&^ All scores are dichotomized (low/high) with reference to high scores.
